# The Epidemiological, Clinical, and Therapeutic Profile of Bipolar Patients in Eastern Morocco

**DOI:** 10.7759/cureus.61769

**Published:** 2024-06-05

**Authors:** Jihane Moussaoui, Ikram Saadi, Mohammed Barrimi

**Affiliations:** 1 Psychiatry, Faculty of Medicine and Pharmacy of Oujda, Mohammed I University, Oujda, MAR; 2 Immunohematology and Cellular Therapy, Faculty of Medicine and Pharmacy of Oujda, Mohammed I University, Oujda, MAR

**Keywords:** childhood trauma, mania, depression, addiction, suicide, bipolar disorder

## Abstract

Introduction: Bipolar disorder is a severe psychiatric disorder. The objective of our study is to describe the epidemiological, clinical, and therapeutic profile of patients followed for bipolar disorder in this hospital.

Materials and methods: This is a cross-sectional, descriptive study conducted over a period of two years within the mental health and psychiatric diseases department of the Mohammed VI University Hospital in Oujda, Morocco, including 206 patients followed for bipolar disorder on an outpatient basis or hospitalized in one of the departments of the hospital.

Results: We included in our study 206 patients with an average age of 37.34+/-12.53 and a male predominance. About 17% of our patients reported a personal medical history and 18.4%, a personal surgical history. Regarding the family history, 40.3% have a medical and surgical history and 63.1%, a psychiatric family history. The consumption of psychoactive substances is present in 49.5% of our patients. About 26.7% reported psychological trauma during childhood. Our patients have reported a history of suicide attempts with a prevalence of 26.6% in several settings: depressive (15%), delusional (5.8%), hallucinatory (3.9%), and anxious (1.9%). Several means were used during these suicide attempts, in particular, defenestration (40%), drug ingestion (36.4%), caustic ingestion (16.4%), strangulation (16.4%), and hanging (10.9%).

Conclusion: It is important to develop a personalized approach to the patient wherever possible and, if necessary, to involve other specialists in diagnosis and treatment.

## Introduction

Bipolar disorder is a severe psychiatric disorder made up of a chronic succession of phases of remission and manic, hypomanic, depressive, or mixed relapses. As with most psychiatric disorders, the precise etiology of bipolar disorder is unknown but probably involves a dynamic interaction between genetic vulnerabilities and several environmental risk factors occurring along a developmental trajectory. It is considered by the World Health Organization to be the sixth leading cause of disability worldwide, associated with a high risk of mortality from suicide, homicide, accident, or medical cause (e.g., cardiovascular disease) [1]. 

Bipolar I disorder is characterized by alternating major depressive episodes and manic phases. Bipolar II disorder presents a course characterized by the appearance of one or more major depressive episodes, accompanied by at least one hypomanic episode [1]. Internationally, the lifetime prevalence of bipolar disorder is 0.5-3.7% [1,2]. In Morocco, according to the national survey conducted in 2003 and published in 2007 by the Ministry of Health studying the prevalence of mental disorders, the prevalence of manic episodes is 3.2% and of major depressive disorder is 26.5% in the general population [3].

Bipolar disorder is a particularly complex illness. It evolves in very different forms, often poorly recognized and poorly treated in clinical practice [1]; hence, there is interest in studies offering an overview of the epidemiological profile of these patients and the particularities of their clinical and therapeutic profile of psychiatric illnesses and mental health at the Mohammed VI University Hospital in Oujda, Morocco. Eastern Morocco is one of the 12 regions of Morocco established by the territorial division of 2015. The density of the population is 28 inhabitants/km^2^, the northern part of the region concentrates nearly 80% of the population of the region, and the rate of urbanization is 67%. The city of Oujda is the capital and has a psychiatric hospital dependent on the Mohammed VI University Hospital with a litter capacity of 108 beds. This hospital provides outpatient and hospital care for patients throughout the region [4]. The objective of our study is to describe the epidemiological, clinical, and therapeutic profile of patients followed for bipolar disorder in this hospital.

## Materials and methods

This is a cross-sectional, descriptive study conducted over a period of two years (2020-2021) within the mental health and psychiatric diseases department of the Mohammed VI University Hospital in Oujda, Morocco, including 206 patients followed for bipolar disorder on an outpatient basis or hospitalized in one of the departments of the hospital.

The data were collected by a single investigator during a psychiatric interview conducted by a resident doctor in psychiatry using a standardized and pre-established exploitation sheet mentioning the socio-demographic data, the antecedents, the clinical profile of the bipolar disorder, its evolution, and the therapeutic profile.

The addictological profile of patients was assessed using several scales. We used the Fagerstrom scale for the evaluation of tobacco addiction, the Cannabis Abuse Screening Test (CAST) questionnaire for the evaluation of cannabis dependence, the Alcohol Use Disorders Identification Test (AUDIT) questionnaire for the evaluation of alcohol consumption, and the Cognitive Scale of Attachment to Benzodiazepines (ECAB) scale for the evaluation of benzodiazepine addition.

Our inclusion criteria were, respectively, consent to participate and age between 17 and 70 years old with a diagnosis of bipolar disorder according to the Diagnostic and Statistical Manual of Mental Disorders, Fifth Edition (DSM-5) criteria, while excluding patients who were very psychomotorly unstable or presented with thymic elements apart from bipolar disorder (recurrent depression/schizoaffective disorder).

Our study also obtained the favorable agreement of the Comité d'Éthique pour la Recherche Biomédicale d'Oujda (CERBO) under order number 19/2020. Statistical analysis was performed using the Epi Info software Version 3.5.1 (Centers for Disease Control and Prevention, Atlanta, Georgia, United States).

The comparison of percentages will be made by the chi-squared test or Fisher's exact test and Student's t-test for the comparison of means. Moreover, our 206 patients all expressed their informed and approved consent during the remission phases of their bipolar disorder.

## Results

We included in our study 206 patients with an average age of 37.34+/-12.53 years and a male predominance with a sex ratio (male-to-female) of 1.39. All the epidemiological characteristics of our sample are presented in Table 1. 

**Table 1:** The epidemiological profile of bipolar patients in eastern Morocco

	Total in % (n=206)
Gender	
Male	120 (58.3%)
Female	86 (41.7%)
Marital status	
Single	96 (46.6%)
Married	78 (37.9%)
Divorced	25 (12.1%)
Widowed	7 (3.4%)
Geographical origin	
Urban	183 (88.8%)
Rural	23 (11.2%)
Professional activity	
Yes	102 (49.5%)
No	104 (50.5%)
Economic level	
Low	100 (48.5%)
Medium	83 (40.3%)
High	23 (11.2%)
Level of education	
Not in school	20 (9.7%)
Primary	48 (23.3%)
Secondary	72 (34.9%)
Bachelor's degree	22 (10.7%)
University	44 (21.4%)

Thirty-five patients (17%) in our sample reported a personal medical history and 38 (18.4%), a personal surgical history. Regarding the family history, 83 (40.3%) patients have a medical and surgical history and 130 (63.1%), a psychiatric family history. The consumption of psychoactive substances is present in 102 patients (49.5%). About 55 patients (26.7%) reported psychological trauma during childhood. All of these antecedents are shown in Table 2. 

**Table 2:** The personal and family history of our bipolar patients

Pathological history	Type	Total in % (n=206)
Personal medical	Diabetes	10 (4.9%)
	Hypothyroidism	8 (3.9%)
	Hyperthyroidism	5 (2.4%)
	High blood pressure	8 (3.9%)
	Heart disease	13 (6.3%)
	Dermatological disorder	7 (3.4%)
Substance use	Cigarettes	102 (49.5%)
	Cannabis	65 (28.6%)
	Alcohol	34 (16.5%)
	Cocaine	3 (1.5%)
	Benzodiazepines	8 (3.9%)
Family medical history	Depression	26 (12.6%)
	Bipolar disorder	88 (42.7%)
	Schizophrenia	17(8.3%)
	Suicide attempt	7 (3.4%)
	Suicide	15 (7.3%)
	Substance use	155 (75.2%)
Psychological trauma in childhood	Sexual abuse	13 (6.3%)
	Parental conflicts	7 (3.4%)
	Death of parents	3 (1.5%)
	Rape	9 (4.4%)
	Physical abuse	26 (12.6%)

The clinical and therapeutic profile of bipolar disorder in our sample is summarized in Table 3.

**Table 3:** The clinical and therapeutic profile of our bipolar patients

	Total in % (n=206)
The clinical profile of bipolar patients	
Type of the first episode	
Manic episode	91 (44.2%)
Depressive episode	75 (36.4%)
Acute psychotic episode	30 (14.6%)
Hypomanic episode	8 (3.9%)
Mixed state	2 (0.9%)
Average age of the first episode	24.81+/-7.64 years
Type of bipolar disorder	
Type I	165 (80.1%)
Type II	41 (19.9%)
The dominant polarity	
Depressive	68 (33.1%)
Manic	138 (66.9%)
Average duration of episodes	4.29+/-18.94 months
Average number of depressive episodes	2.28+/-3.36
Average number of manic episodes	2.70+/-3.40
Average number of hypomanic episodes	0.90+/-1.91
Average number of mixed episodes	0.06+/-0.26
History of psychiatric hospitalization	
Yes	185 (89.8%)
No	21 (10.2%)
Average number of psychiatric hospitalizations	2.82+/-2.68
Average length of hospitalization	29.95+/-15.51 days
Average age of first hospitalization	29.44+/-10.41 years
Average duration of disease progression	12.13+/-10.67 years
Average time between the onset of the disease and its diagnosis	4.28+/-6.58 years
The therapeutic profile of bipolar patients	
Average age of first treatment in years	26.39+/-9.66 years
Carbamazepine	
Yes	34 (16.5%)
No	172 (83.5%)
Sodium valproate	
Yes	95 (46.1%)
No	111 (53.9%)
Quetiapine	
Yes	16 (7.8%)
No	190 (92.2%)
Lamotrigine	
Yes	5 (2.4%)
No	201 (97.6%)
Atypical neuroleptics	
Yes	144 (69.9%)
No	62 (30.1%)
Classical neuroleptics	
Yes	9 (4.4%)
No	197 (95.6%)

Bipolar disorder is also the psychiatric disorder most likely to cause suicidal behavior, so 55 of our patients have reported a history of suicide attempts (26.6%) in several settings: depressive in 31 patients (15%), delusional in 12 patients (5.8%), hallucinatory in eight patients (3.9%), and anxious in four patients (1.9%). Several means were used during these suicide attempts, in particular, defenestration in 82 patients (40%), medication ingestion in 75 patients (36.4%), caustic ingestion in 34 patients (16.4%), strangulation in 34 patients (16.4%), and hanging in 22 patients (10.9%).

## Discussion

Bipolar disorder is estimated to affect approximately 3% of the general population according to the American Psychiatric Association (2013) [5]. Although most epidemiological studies have identified that bipolar I disorder affects approximately 1% of the population adults, more recent work suggests that up to 4% more may be affected by bipolar spectrum disorders such as bipolar II disorder and cyclothymia. Bipolar I disorder affects an equal proportion of men and women, but type II seems to be more common in women [6]. The average age of onset is 18.2 years for bipolar I disorder and 20.3 years for bipolar II disorder. The time between the first symptoms and the establishment of a correct diagnosis is particularly long (on average 10 years). The most common "erroneous" diagnosis is that of unipolar depression, particularly for bipolar II disorder [7,8].

A meta-analysis of 14 epidemiological studies (regrouping 62,736 subjects) was recently carried out reporting a lifetime prevalence of bipolar I disorder in adults estimated on average at 0.82% (95% CI: 0.42-1.21). Then, a recent literature review showed that the annual cost of bipolar disorder is between €10,000 and €16,000, of which 80% are indirect costs, 15% are related to hospitalization, and 5% are related to contrast medication [7,8]. With an overall low socioeconomic level for the majority of our bipolar patients, most people with bipolar disorder typically experience depression as their first mood episode. Treated depressive episodes have a median duration of 15 weeks, while the median duration is seven weeks for manic episodes and three weeks for hypomania according to one study. Shorter periods of euthymia are associated with poorer functioning, so identifying predictors of relapse is crucial to aid in treatment [8].

Depression is the most disabling phase of the disease and the most associated with the risk of morbidity and mortality. Depression is also the most apparent syndrome in the disease, with mania or hypomania being rarely reported spontaneously by the patient as an abnormal period of his life. About 40% of patients with bipolar disorder experience mixed episodes, defined as a manic state with depressive features or manic symptoms in a patient with bipolar depression [8]. Compared to bipolar patients without mixed features, patients with bipolar mixed states generally have more severe symptomatology, more lifetime episodes of illness, poorer clinical outcomes, and higher rates of comorbidities and therefore present a significant clinical challenge [8,9]. Major depressive disorder has a strong genetic component associated with its etiology. Genetic studies have found a 37% heritability and a 2.8-fold increased risk of developing the condition in children of first-degree relatives with major depressive disorder [9].

Bipolar disorder is rarely an isolated condition, comorbidity rather being the rule, notably with anxiety disorders and the use of psychoactive substances and personality disorders. Systematic reviews suggest that borderline personality disorder is present in about 20% of people with bipolar disorder, with higher rates of bipolar II disorder compared to bipolar I disorder. Anxiety disorders are the most common, with nearly 60% of patients with bipolar disorder meeting the criteria for one of these conditions, while substance use disorders are seen in as many as 24% of patients with bipolar disorder. Bipolar disorder is often associated with other psychiatric disorders. These can modify the clinical presentation of bipolar disorder and make the diagnosis sometimes difficult. Physical comorbidities are also generally present, most often metabolic, cardiovascular, thyroid, and neurological [10,11]. Suicide accounts for nearly 20% of all deaths in people with bipolar disorder, and between 25% and 50% will attempt suicide [10,11]. Suicidal behaviors in bipolarity occur more frequently during an inaugural depressive episode and in the case of long-duration evolution of the disease without treatment [12] as well as in the case of early onset of the disease, personal or family history of attempted suicide or suicide, and then psychiatric comorbidities such as the use psychoactive substances and anxiety disorders [13]. Other studies report a frequency of suicidal behavior during the depressive, mixed phase, rapid cycles or if there are psychotic elements present, and the only treatment that has proven its anti-suicidal efficacy is lithium [14]. Bipolar disorder requires the pharmacological management of acute depressive and acute manic episodes and prophylactic treatment against relapses. Anti-convulsing drugs such as sodium valproate and carbamazepine are also widely prescribed as mood stabilizers. They are less effective in the prevention of long-term relapses. All surveys found that mood stabilizers are underused and that overall only one in four patients with bipolar disorder would receive adequate treatment. Atypical antipsychotics clearly have acute antimanic efficacy, but their long-term preventive efficacy is less clearly established [15]. The Food and Drug Administration guidance now exists for olanzapine and aripiprazole in the maintenance treatment of bipolar disorder, but these data are quite limited, with the study on aripiprazole being rather short (six months) and the study on olanzapine limited by a high rate of acute relapse withdrawal in the placebo arm, thus demonstrating short-term rather than long-term withdrawal. Most authors agree that treatment delay in bipolar patients is related to poor social adjustment, higher number of hospitalizations, increased risk of suicide, development of comorbidities, medico-legal complications, and overall impaired ability to cope with developmental tasks in bipolar patients [15]. In a recent retrospective study, bipolar patients experienced more life events, including losses, in the year preceding their first depressive episode or their first manic episode, compared to healthy subjects. In addition, more than 60% of first depressive episodes are preceded by at least one stressful event, whereas this rate is less than 20% in the general population. This influence of life events in the induction of a disorder mood is more important in the early stages than in the late stages of bipolar disorder [15].

## Conclusions

Bipolar disorder is a mood disorder with a considerable impact on the quality of life of patients and their families. Psychiatric and physical comorbidities must be treated in parallel with the underlying psychopathology, as they can worsen the overall clinical picture, increase the risk of re-hospitalization, and hamper the therapeutic management of patients with bipolar disorder. It is therefore important to develop a personalized approach to the patient wherever possible and, if necessary, to involve other specialists in diagnosis and treatment.
